# Adaptation of Parasocial Relationships in Social Media Scale: its association with loneliness, social media use and life satisfaction

**DOI:** 10.3389/fpsyg.2026.1814389

**Published:** 2026-09-17

**Authors:** Idil Eren Kurt, Fatoş Bulut-Ateş, Hacı Arif Doğanülkü

**Affiliations:** Department of Psychological Counseling and Guidance, Çukurova University, Adana, Türkiye

**Keywords:** life satisfaction, loneliness, parasocial relationships, PRISM-TR, social media, social media use

## Abstract

The advancement of digital technologies, particularly social media, has significantly altered the dynamics of connection formation, making the study of parasocial relationships highly relevant. This study analyzed the psychometric properties of the Parasocial Relationships in Social Media Scale (PRISM-TR) within a Turkish sample group. The research involved two distinct sub-studies utilizing different samples. Study I (*N* = 428 university students) employed comprehensive psychometric methods, including Confirmatory Factor Analysis (CFA), Item Response Theory (IRT), and reliability analyses, to support the scale’s four factor structure, internal consistency, and measurement invariance across gender. Study II (*N* = 674 students) investigated the interplay between the variables by testing a serial mediation model. The findings revealed that loneliness significantly predicts parasocial relationships, a connection that is serially mediated by social media use and life satisfaction. Collectively, these results validate the PRISM-TR scale’s effectiveness for evaluating parasocial relationships in this context while simultaneously highlighting the complex sequential influence of digital media engagement and life satisfaction factors in shaping these modern bonds. The findings carry important implications for psychological counseling and digital literacy programs targeting young adults.

## 1 Introduction

The significant development of digital technologies and the widespread use of social media have brought about profound changes in how individuals form, maintain, and experience social relationships. In this framework, parasocial relationships originally conceptualized by Horton and Wohl (1956) refer to unidirectional bonds where individuals experience emotional attachment despite the absence of mutual interaction. Social media platforms offer a different level of social relatedness than traditional media environments by enabling individuals not only to consume content but also to be active participants and interactors (Marwick and Boyd, 2011). Within this environment, connections with media figures have gone beyond traditional parasocial relationships, becoming more accessible, continuous, and emotionally intense. Today, individuals can experience media figures as part of their social circles, and their parasocial relationships with these figures can appear to be bidirectional interactions due to the nature of social media (Labrecque, 2014; Chung and Cho, 2017). This transformation has necessitated new approaches to understanding the motivations behind social media use.

Numerous qualitative and quantitative studies have examined parasocial relationships in conjunction with individual and social variables such as loneliness (Giles, 2002; Hall, 2025; Liu and Lee, 2024; Lotun et al., 2024), social anxiety (Tatem and Ingram, 2022; Jia et al., 2023), self-esteem (Chauvin et al., 2024; Paravati et al., 2024), life satisfaction (Krouse, 2024; Lachmann et al., 2018), frequency of media use (Tatem and Ingram, 2022; Tukachinsky and Stever, 2019), digital self-efficacy (Kim et al., 2024), emotion regulation difficulties (Hartmann and Goldhoorn, 2011), attachment (Giles and Maltby, 2004), and motivations for social media interaction (Chen and Xiao, 2022; Paravati et al., 2020); related research has also extended parasocial relationship frameworks to emerging digital interaction settings, including chatbot-based environments (e.g., Ramya and Alur, 2025). In addition, tendencies for social comparison (Chung and Cho, 2017) and interpersonal relationship difficulties (Abdinagoro and Bismo, 2025; Rubin and McHugh, 1985) have been associated with the formation and depth of parasocial bonds. These findings emphasize the relevance of social and emotional needs in the formation of parasocial bonds. Furthermore, longitudinal studies exploring how parasocial relationships develop over time and accompany social experiences have shed light on their dynamic nature (Bond, 2021a,b).

Existing scales used to assess parasocial relationships such as the Parasocial Interaction Scale (PSI) (Rubin et al., 2020), the Audience Persona Interaction Scale (API) (Auter and Palmgreen, 2000), and the Celebrity Persona Parasocial Interaction Scale (CPPI) (Bocarnea, 2007) were typically developed for traditional media environments like television and radio, often structured around newscasters or fictional characters. These scales fall short of capturing the interactive nature and dynamic characteristics of digital platforms, limiting their effectiveness in measuring parasocial relationships in social media contexts. To address this need, the Parasocial Relationship in Social Media (PRISM) scale (Boyd et al., 2022, 2024) was developed as a context specific instrument designed to assess parasocial relationships formed on social media. While the Turkish literature includes a scale titled the “Scale of Being Influenced by Social Media Phenomena” (Tagay et al., 2024), it primarily targets adolescents and focuses on the impact of social media figures on personality development, approaching the topic from a popular culture perspective. Rather than examining the concept of parasocial relationships through a theoretical lens, this scale aims to measure the influence of social media figures on young people’s lifestyle and value perceptions. In contrast, the PRISM scale is grounded in a conceptual framework that seeks to assess parasocial relationships in terms of communicative connection, perceived intimacy, and unidirectional interaction with content creators. Therefore, adapting the PRISM scale into Turkish within the current study provides a theoretically coherent examination of one-sided relationships developed through social media engagement, providing a robust foundation for both theoretical and applied research. Moreover, the availability of this measurement tool in Turkish offers researchers a valid and reliable means to explore the psychological dimensions of social media interactions.

Empirical findings on parasocial relationships can be interpreted within broader theoretical frameworks that help explain why and how individuals form bonds in digital environments. The Compensatory Internet Use Theory (Kardefelt-Winther, 2014) offers a relevant theoretical framework in this regard. According to this theory, individuals turn to the internet not because of the technology itself, but as a way of coping with adverse life circumstances or unmet psychosocial needs. In the present study, this framework is drawn upon specifically to conceptualize why individuals experiencing loneliness may increasingly turn to social media, rather than to account for the full sequence leading to parasocial relationship formation. Although such engagement may provide emotional relief in the short term, over time it may reduce social satisfaction and foster greater reliance on virtual connections. In this way, social media becomes not merely a source of entertainment but also a space in which individuals attempt to meet unmet psychosocial needs.

Empirical studies based on this theory have shown that individuals may turn to the internet to compensate for difficulties experienced in offline life. Kuss et al. (2013) found that social anxiety and low self-perception are associated with increased time spent on social media, suggesting a compensatory motive. Dempsey et al. (2019) highlighted that social isolation is associated with more intensive engagement with platforms like Instagram, where such use may temporarily reduce loneliness but potentially lead to digital addiction in the long term. Elhai et al. (2020) indicated that elevated stress and anxiety symptoms are linked to problematic social media use, reflecting a potential emotion regulation strategy. These findings suggest that individuals tend to meet their psychosocial needs through social media and that this usage is closely linked to feelings such as loneliness, stress, and low life satisfaction.

Explaining parasocial relationships solely through compensatory motivations such as loneliness and social deprivation captures only a partial picture of their function in digital contexts, particularly for emerging adults. For the demographic focused on in this study, university students, a core psychosocial developmental task is identity formation (Arnett, 2000). Typically, identity is constructed through active social engagement and peer validation. However, when individuals experience high levels of loneliness, their offline avenues for peer-based identity exploration are restricted. This social deficit, and the resulting struggle to establish a coherent sense of self within a community, can significantly diminish an individual’s overall life satisfaction. In response to these unmet offline needs, social media constitutes a particularly potent environment for alternative exploration: content creators, influencers, and celebrities function as idealized models through which individuals acquire culturally transmitted knowledge pertaining to status, achievement, and identity construction (Butler, 2024).

Viewed through this lens, parasocial relationships formed on social media may represent more than just passive substitutes for real world interpersonal bonds, they can also function as active psychological processes through which dissatisfied and isolated individuals might attempt to safely construct their identities. Consistent with this perspective, Nesi and Prinstein (2019) demonstrated that adolescents invest considerable effort in accumulating online indicators of peer status such as likes and followers and that this orientation toward digital status seeking is directly associated with intensified social media engagement. Collectively, these dynamics suggest that parasocial relationships in digital contexts are shaped by a complex interplay of motivations: they serve to compensate for the emotional toll of loneliness and low life satisfaction, while simultaneously functioning as risk-free channels for identity formation. Accordingly, examining loneliness, social media use, life satisfaction, and parasocial relationships within a single integrative model constitutes a theoretical necessity for capturing how digital media use addresses both emotional deficits and developmental needs.

Loneliness is a significant determinant in the development of parasocial relationships. Studies have shown that individuals who experience social exclusion, rejection, or persistent loneliness attempt to meet their social needs through parasocial connections with media figures (Stein et al., 2024; Lotun et al., 2024). For lonely individuals, such relationships provide a safer alternative to real life connections by avoiding the disappointments often associated with them. Accordingly, loneliness appears to increase the tendency to engage in parasocial relationships (Baek et al., 2013; Liu and Lee, 2024). As previously stated, challenges in fulfilling social needs relate to higher levels of social media engagement and a greater inclination to seek stimulation through these digital spaces (Bessiere et al., 2008; Bonsaksen et al., 2021; Chura-Quispe et al., 2025; Longstreet and Brooks, 2017; Nowland et al., 2018; Pittman and Reich, 2016; Satici, 2019; Wright and Riviera, 2025; Yang, 2016). Therefore, social media use may serve as a mediating mechanism in the relationship between loneliness and parasocial relationships by allowing individuals to fill their social void through connections with media figures.

Conversely, numerous scholarly works have shown that lonely individuals tend to report lower life satisfaction (Arampatzi et al., 2018; Best et al., 2021; Çivitci and Çivitci, 2009; Heinrich and Gullone, 2006; Salimi, 2011; Satici, 2019; Tu and Zhang, 2015). Loneliness is associated with lower levels of satisfaction in interpersonal relationships and more negative evaluations of life in general (Qualter et al., 2015). As a result, individuals may seek to compensate for unmet social needs through alternative means. At this point, parasocial relationships may be understood as serving a compensatory function. According to the parasocial compensation hypothesis (Madison et al., 2016), when individuals do not derive sufficient satisfaction from their real-life interactions, they may be inclined to form one-sided bonds with media figures in order to compensate for unmet social and emotional needs. Because life satisfaction reflects an individual’s global, cognitive-evaluative judgment of their life (Diener et al., 1985), lower life satisfaction may plausibly be associated with such a compensatory orientation; indeed, parasocial relationships can provide low-risk, accessible, and emotionally safe connections that may facilitate this compensatory process (Giles, 2002; Stever, 2024).

It should be noted, however, that much of the empirical work in this area relies on closely related constructs—such as subjective well-being (Krouse, 2024) or belongingness needs (Greenwood and Long, 2009)—rather than life satisfaction as such. Nevertheless, the direct empirical relationship between life satisfaction and parasocial relationships has been examined only to a limited extent, and the available findings are complex and inconsistent. Moreover, most studies in this area do not assess life satisfaction directly, but rather broader constructs such as subjective or psychological well-being; for instance, Ravi and Patki (2025) found no significant association between the intensity of parasocial relationships and psychological well-being. Given that life satisfaction constitutes only the cognitive-evaluative component of subjective well-being (Diener et al., 1985), the generalizability of such findings to the specific life-satisfaction variable examined in the present study is limited. For this reason, the pathway from life satisfaction to parasocial relationships is approached in the present study as a theoretically grounded yet exploratory link, rather than as a definitive causal claim.

Existing literature also points to a negative link between social media use and life satisfaction, often involving factors such as increased social comparison, fear of missing out (FoMO), low self esteem, and feelings of loneliness (Appel et al., 2016; Hall et al., 2021; Ozimek et al., 2024; Pahlevan-Sharif et al., 2025; Shahnaz and Karim, 2014; Verduyn et al., 2017; Vogel et al., 2014). In sum, the multifaceted nature of individuals’ social interaction experiences in digital environments necessitates the joint examination of the four variables addressed in this study.

### 1.1 Current study

The research consists of two separate studies with two separate samples. Study 1 involves the adaptation of the PRISM scale, with the primary goal of establishing its validity and reliability. Following the Turkish adaptation of the scale, a model testing study was conducted (Study 2). Building on the significant relationship between loneliness and parasocial relationships, this study focuses on the serial mediating role of social media use and life satisfaction. Taking the Compensatory Internet Use Theory as a foundation to explain the initial drive to use digital tools (e.g., social media) to cope with negative psychosocial states such as loneliness, this study expands upon this link by examining loneliness, life satisfaction, and parasocial relationships together to understand how individuals fulfill their social needs in digital contexts. Specifically, it is proposed that individuals experiencing higher levels of loneliness may be more inclined toward greater social media use; that this intensified engagement may in turn be associated with diminished life satisfaction; and that lower levels of life satisfaction may be linked to a heightened tendency to emotionally engage with media figures through parasocial relationships.

In line with this theoretical rationale, the serial mediation model proposed in the present study is grounded in a theoretically coherent and empirically supported sequence of mechanisms. Although prior research has examined these variables in dyadic combinations linking loneliness to social media use (Bonsaksen et al., 2021; Nowland et al., 2018), social media use to life satisfaction (Appel et al., 2016; Verduyn et al., 2017), the direct link between life satisfaction and parasocial relationships has received comparatively limited and inconsistent empirical attention, and integrative models that simultaneously encompass all four variables remain insufficiently developed in the literature. This represents a critical gap, as the isolated examination of dyadic associations fails to capture the cumulative and cascading nature of the psychological processes through which loneliness ultimately gives rise to parasocial relationship formation in digital contexts. It should be emphasized that treating this pathway as exploratory does not preclude specifying a theoretically justified direction for the model. Rather, the proposed ordering reflects a modeling rationale rather than a claim of established causality, and it is consistent with the input–process–output logic. In our framework, life satisfaction functions as a global and comparatively stable cognitive evaluation (Diener et al., 1985), rather than a transient state shaped solely by isolated media interactions. Within this logical sequence, broad cognitive evaluations (life satisfaction) tend to precede and inform specific behavioral or relational outcomes (engagement in parasocial relationships). Positioning parasocial relationships as a predictor of global life satisfaction would contradict this conceptualization; accordingly, life satisfaction is modeled as an antecedent predicting parasocial engagement, rather than as its outcome.

By testing these pathways simultaneously within a single structural model, the present study moves beyond descriptive associations to offer a theoretically driven explanation of the mechanisms underlying parasocial relationship formation on social media. In doing so, this study extends the Compensatory Internet Use Theory beyond its conventional application to problematic internet use, bringing it to bear on the formation of parasocial relationships in digital contexts and proposing that compensatory social media use may be embedded within a sequential psychological process with broader implications for individuals’ social and emotional lives. It further proposes that life satisfaction may operate as an intermediate variable linking loneliness-driven social media use to parasocial relationship formation—a pathway that has yet to be empirically examined within an integrative model.

Within this framework, the primary aim of this study is twofold: first, to adapt and validate the Parasocial Relationships in Social Media Scale (PRISM) for the Turkish context; and second, to examine the interrelations among loneliness, social media use, life satisfaction, and parasocial relationships using a structural equation model, thereby offering a deeper understanding of the psychological foundations of individuals’ social interactions in online settings.

This study proposes the following hypotheses:

*H1*: The PRISM-TR is a psychometrically valid and reliable tool in Turkish.

*H2*: Loneliness positively predicts parasocial relationships.

*H3*: Social media use mediates the relationship between loneliness and parasocial relationships.

*H4*: Life satisfaction mediates the relationship between loneliness and parasocial relationships.

*H5*: Social media use and life satisfaction serially mediate the relationship between loneliness and parasocial relationships.

## 2 Study I

In Study I, the Parasocial Relationships in Social Media Scale (PRISM), developed by Boyd et al. (2022, 2024), was adapted into Turkish. PRISM was evaluated and translated into Turkish by four academicians who are fluent in English and native speakers of Turkish. To ensure the accuracy of the translation with the original structure, the back-translation method was employed (Brislin, 1970). In this process, the initial Turkish translation was independently translated back into English by a bilingual expert who had not seen the original scale. The original and back-translated versions were then compared to resolve any discrepancies and finalize the phrasing. A sample item illustrating this translation process is provided in Supplementary Material 1, and the complete final Turkish version of the scale (PRISM-TR) is provided in Supplementary Material 2 to facilitate its use in future research. Subsequently, the PRISM-TR scale was administered online to participants in the Turkish sample, and data collection was completed. At the outset of the research, a confirmatory factor analysis (CFA) was performed to examine the scale’s factorial structure. This was followed by an examination of item total correlations and analyses based on Item Response Theory (Baker, 2001; Harwell et al., 1996). The phase concluded with reliability testing.

### 2.1 Method

#### 2.1.1 Participants

Data were collected online, and written informed consent was obtained voluntarily from all participants prior to participation. Study 1 data were collected from 428 university students. Among the participants, 285 (66.6%) identified as female and 143 (33.4%) as male. The mean age was 21.18 years (SD = 2.11), with ages ranging from 18 to 32.

#### 2.1.2 Procedure

Data collection was conducted through an online survey platform (Google Forms). The survey link was distributed to university students through university communication channels and social media groups using convenience sampling. Prior to accessing the survey items, all participants were presented with an informed consent page detailing the purpose of the study, the voluntary nature of participation, and data confidentiality. Participants who provided digital consent proceeded to the questionnaires. The completion of the survey took approximately 10–15 min. The questionnaires were presented in a fixed, non-counterbalanced order for all participants (i.e., demographic questions followed by PRISM-TR, PSBASMI, and SOLSMIEF), as the primary goal was to validate the PRISM-TR scale and the platform did not utilize automated block randomization.

#### 2.1.3 Measures

In this section, the measurement instruments used in Study I are introduced.

##### 2.1.3.1 Parasocial relationships in social media scale (PRISM)

The Parasocial Relationships in Social Media (PRISM) scale was developed by Boyd et al. (2022, 2024) to assess individuals’ parasocial relationships on social media. The scale is composed of 22 items distributed across four subscales: Interest In (7 items) evaluates the interest in the creator’s content, valuing their goals, and the desire to follow their content; Knowledge Of (5 items) measures active information seeking about the content creator, awareness of their personal life, and the willingness to learn everything about them; Identification With (6 items) assesses the feeling of shared beliefs, opinions, and attitudes with the content creator, as well as alignment with their decisions and adopting their ways of solving problems; and Interaction With (4 items) measures feeling as if with the content creator as a friend, the sense of not being alone in their presence, and the feeling of establishing an intimate bond. The scale employs a five point Likert format, ranging from “strongly disagree” to “strongly agree.” Increasing scores indicate a higher level of parasocial involvement on social media. There are no reverse scored items in the scale. Factor loadings in the original 22 item structure of the PRISM range between 0.52 and 0.86.

##### 2.1.3.2 Perception scale of being affected by social media influencers (PSBASMI)

The scale was developed by Tagay et al. (2024) to determine the extent to which individuals are affected. It is one dimensional and consists of eight items. The five point Likert type scale offers options between “Not at all suitable” and “Completely appropriate.” There are no reverse scored items in the scale. The increase in the scores obtained from the scale indicates an increase in being influenced by social media phenomena. Analyses of the original form of the scale revealed a good fit (*χ*^2^*/*d*f* = 1.69, RMSEA = 0.08, CFI = 0.98, NFI = 0.95, NNFI = 0.97, GFI = 0.93, and AGFI = 0.87). Cronbach’s alpha coefficient, calculated within the scope of the reliability analysis of the scale, was calculated as 0.85.

##### 2.1.3.3 The scale of opinion leadership of social media influencers in the eyes of followers (SOLSMIEF)

It was developed by Tam (2020) to assess the extent to which individuals are influenced by social media phenomena. The scale consists of six dimensions: knowledge, closeness, imitation, trust, communication, and entertainment, and a total of 22 items. The five point Likert type scale offers options between “strongly disagree” and “strongly agree.” There are no reverse scored items in the scale. An increase in scores on the scale indicates that individuals are influenced by social media phenomena. When the CFA results performed within the scope of the validity study of the scale were examined, it was determined that the model fit was excellent (*χ*^2^/d*f* = 2.735; NFI = 0.91; AGFI = 0.89; RFI = 0.96; NNFI = 0.93; GFI = 0.91; CFI = 0.91; RMSEA = 0.05). When the reliability analysis conducted by Tam (2020) was examined, the Cronbach’s alpha internal consistency coefficients were found to range between 0.77 and 0.87.

#### 2.1.4 Data analysis

The preliminary descriptive analyses were carried out using IBM SPSS Statistics version 26. Subsequently, the 22 item structure of PRISM-TR was subjected to Confirmatory Factor Analysis (CFA) via AMOS Graphics to assess its factorial validity. In addition, structural, metric, and scalar invariance tests were performed to evaluate whether the scale functioned equivalently across genders. The item discrimination and difficulty parameters of the scale were assessed through Item Response Theory (IRT) analysis performed in Stata. To determine the internal consistency and reliability of the scale, several coefficients were calculated, including Cronbach’s Alpha (*α*), McDonald’s Omega (*ω*), and Guttmann’s Lambda 6 (*λ6*).

### 2.2 Results

The psychometric properties of the 22 item, four dimensional form of the scale were examined, and it was observed that it showed an acceptable model fit (*χ*^2^/d*f* = 3.49; CFI = 0.93; NFI = 0.90; TLI = 0.90; RMSEA = 0.076). The standardized factor loadings of the items of the scale ranged between 0.63 and 0.89, and all of them were found to be significant (*p* < 0.001). Factor loadings, item total correlations, and statistics are presented in Table 1.

**Table 1:** Factor loadings, descriptive statistics and item-total correlations.

Factor	Item	Factor loadings	Mean	SD	Item total correlations
F1 (interest)	1. I look forward to seeing [NAME]‘s content.	0.75	2.25	1.06	0.69
	2. I like to see content that [NAME] has made.	0.72	3.10	1.14	0.69
	3. I am interested in [NAME].	0.75	2.90	1.13	0.71
	4. I care about what happens to [NAME].	0.70	2.05	1.07	0.71
	5. I hope [NAME] achieves their goals.	0.63	3.07	1.17	0.62
	6. I would follow [NAME] on another account if they create one on the same social media site that I already follow them on.	0.76	2.49	1.21	0.72
	7. I like to see content that [NAME] is in that they did not make themselves.	0.74	2.22	1.13	0.70
F2 (knowledge)	1. When I come across information about [NAME], I will search to learn more about them.	0.82	2.15	1.13	0.73
	2. I seek out information to learn more about [NAME].	0.75	1.81	0.97	0.66
	3. Learning about [NAME] is important to me.	0.74	1.77	0.94	0.68
	4. I am aware of the personal details of [NAME]‘s life.	0.72	2.01	1.07	0.67
	5. If I saw a story about [NAME], I would read it.	0.67	2.84	1.23	0.69
F3 (identification)	1. I have many of the same beliefs as [NAME].	0.77	1.85	1.02	0.72
	2. I have many of the same opinions as [NAME].	0.83	2.02	1.02	0.76
	3. I usually agree with [NAME].	0.86	2.35	1.09	0.76
	4. I usually make the same choices as [NAME].	0.81	2.03	0.98	0.74
	5. I can relate to [NAME]‘s attitudes.	0.83	2.33	1.09	0.77
	6. I like the way [NAME] handles problems.	0.78	2.63	1.18	0.76
F4 (interaction)	1. [NAME] makes me feel as if I am with a friend.	0.88	2.38	1.25	0.77
	2. [NAME] keeps me company when viewing their content.	0.89	2.28	1.25	0.76
	3. I can imagine myself as [NAME]‘s friend.	0.80	2.05	1.16	0.72
	4. [NAME] understands the kinds of content I want to see.	0.68	2.07	1.11	0.70

#### 2.2.1 Measurement invariance

Following the confirmation of the PRISM’s factor structure through confirmatory factor analysis, further analyses were conducted to assess whether the scale measured constructs equivalently across groups. To this end, CFA was applied separately to data from female (*χ*^2^/d*f* = 2.810, TLI = 0.913, RMSEA = 0.080, CFI = 0.925) and male participants (*χ*^2^/d*f* = 1.962, TLI = 0.898, RMSEA = 0.082, CFI = 913). The results indicated an acceptable model fit for both groups. Moreover, findings demonstrated that PRISM-TR met the criteria for both metric (ΔCFI = 0.001) and scalar invariance (ΔCFI = 0.002), as shown in Table 2, suggesting that the construct is interpreted similarly by men and women. Table 3 presents the factors of PRISM-TR and their associations with other variables.

**Table 2:** Fit indices of gender invariance.

Invariance	*χ* ^2^	d*f*	*χ*^2^/d*f*	TLI	ΔTLI	RMSEA	CFI	ΔCFI
Females	556.432	198	2.810	0.913	—	0.080	0.925	—
Males	386.457	197	1.962	0.898	—	0.082	0.913	—
Configural invariance	1076.754	402	2.678	0.889	—	0.063	0.903	—
Metric invariance	1091.386	420	2.599	0.894	0.005	0.061	0.904	0.001
Scalar invariance	1125.033	438	2.569	0.896	0.002	0.061	0.902	0.002

**Table 3:** Descriptive statistics and correlations with PRISM-TR.

Variables	Mean	SD	Skewness	Kurtosis	Correlation with PRISM-TR	
					*R*	*p*
PRISM	50.77	17.67	0.16	−0.94	—	—
PSBASMI	16.82	5.95	0.36	−0.74	0.74	<0.001
SOLSMIEF	45.53	15.67	0.39	−0.61	0.74	<0.001

#### 2.2.2 Item response theory

Item Response Theory (IRT) offers a robust and widely used framework for examining item level response patterns and is applicable to a variety of measurement contexts (Harwell et al., 1996). Unlike classical test theory, which focuses on total scores, IRT emphasizes the evaluation of individual item characteristics (Baker, 2001). For Likert type scales, IRT considers the ordered nature of response categories and models how respondents select among them in a sequential manner (Fraley et al., 2000). Given the ordinal structure of the PRISM-TR, applying IRT was considered appropriate for assessing item level discrimination more precisely. Specifically, the Graded Response Model (GRM) was employed, as it is suitable for analyzing polytomous items. GRM generates estimates for item discrimination (*α*) and category thresholds (b1, b2, b3), offering a nuanced understanding of how each item functions across different levels of the latent trait parasocial relationship tendencies. Based on the criteria proposed by Baker (2001), *α* values exceeding 1.0 reflect strong discriminatory power. In the present analysis, all items demonstrated α values above this threshold (see Table 4).

**Table 4:** IRT results for the PRISM-TR.

Item	〈 coefficient	SE	Confidence interval	*z*	*p > |z|*
Item 1	1.86	0.16	1.54–2.18	11.54	0.000
Item 2	1.84	0.14	1.55–2.13	12.34	0.000
Item 3	1.92	0.15	1.61–2.22	12.40	0.000
Item 4	2.05	0.18	1.70–2.41	11.30	0.000
Item 5	1.44	0.13	1.19–1.70	11.12	0.000
Item 6	2.01	0.16	1.68–2.34	12.03	0.000
Item 7	1.98	0.17	1.64–2.32	11.46	0.000
Item 8	2.19	0.18	1.82–2.55	11.66	0.000
Item 9	1.98	0.18	1.62–2.35	10.62	0.000
Item 10	2.13	0.20	1.74–2.53	10.55	0.000
Item 11	1.81	0.16	1.48–2.14	10.75	0.000
Item 12	1.81	0.15	1.52–2.11	12.00	0.000
Item 13	2.80	0.24	2.31–3.28	11.25	0.000
Item 14	2.96	0.25	2.47–3.46	11.85	0.000
Item 15	2.74	0.22	2.30–3.18	12.27	0.000
Item 16	2.65	0.22	2.21–3.09	11.83	0.000
Item 17	2.75	0.22	2.31–3.19	12.36	0.000
Item 18	2.49	0.19	2.10–2.88	12.65	0.000
Item 19	2.68	0.21	2.26–3.11	12.33	0.000
Item 20	2.77	0.22	2.32–3.22	12.13	0.000
Item 21	2.40	0.20	2.00–2.81	11.61	0.000
Item 22	2.23	0.19	1.85–2.61	11.57	0.000

As presented in Table 4, each item’s discrimination parameter (*α*) exceeded 1.0, indicating a high capacity to differentiate individuals based on their standing on the underlying construct (Baker and Kim, 2017). This suggests that the Turkish adaptation of the PRISM scale successfully captures differences in individuals’ parasocial engagement on social media. Moreover, the statistically significant z values further support the precision and stability of these parameter estimates.

#### 2.2.3 Reliability

Cronbach’s Alpha, McDonald’s Omega, and Guttmann’s Lambda reliability analyses were performed for PRISM-TR. In addition, 104 participants in the group from whom data were collected for CFA were measured again after 3 weeks, and reliability analysis was performed for PRISM-TR using the test retest method. The findings of the reliability analyses are displayed in Table 5. These results indicate that the scale demonstrates satisfactory internal consistency and offers a dependable measurement.

**Table 5:** Reliability results of PRISM-TR.

Reliability coefficient	Value
Cronbach’s alpha	0.95
McDonald’s omega	0.95
Gutmann’s lambda	0.96
Test–retest correlation	0.72

## 3 Study II

Following the evaluation of PRISM-TR’s psychometric properties, Study II examined whether social media use and life satisfaction sequentially mediated the relationship between loneliness and parasocial relationships in the context of social media.

### 3.1 Methods

#### 3.1.1 Participants and procedure

Study II data were collected from 674 university students volunteering to participate in the research. 438 of the participants were female (65%), and 236 were male (35%). The mean age of participants was 20.94 years (SD = 2.81; range = 18–33). Data were collected online using Google Forms. Participants were recruited via university networks and social media platforms. Written informed consent was obtained digitally from all participants before they could access the survey items. The survey battery took approximately 15 min to complete. To maintain consistency, the questionnaires were presented in a fixed order without counterbalancing (demographic information followed by BSMAS, ULS, SLS, and PRISM-TR). Participants were required to complete all items to submit the form, thereby preventing missing data.

#### 3.1.2 Measures

In this section, the measurement instruments used in Study II are introduced.

##### 3.1.2.1 Bergen social media addiction scale (BSMAS)

BSMAS, developed by Andreassen et al. (2016), was adapted into Turkish by Demirci (2019). The scale is one dimensional and consists of six items. The scale is in the form of a five point Likert and offers options between “very rarely” and “quite often.” Higher scores reflect increased levels of social media use. When the confirmatory factor analysis findings of the scale were analyzed with data collected from adults, it was found to have an acceptable level of fit (*χ*^2^*/*d*f* = 1.33, CFI = 0.99; TLI = 0.98; SRMR = 0.039; RMSEA = 0.046). The Cronbach Alpha value of the scale was found between 0.80 and 0.84 in applications with adults.

##### 3.1.2.2 The satisfaction with life scale (SLS)

It was developed by Diener et al. (1985). The validity and reliability studies of the scale adapted to Turkish by Köker (1991) were repeated by Dağlı and Baysal (2016). In Dağlı and Baysal's (2016) study, the scale has a single dimension, five items, and a five point Likert type, and the scores that can be obtained from the scale vary between 5 and 25. Higher scores reflect greater life satisfaction. The internal consistency coefficient of the scale was calculated as 0.88. The scale offers options between “I completely disagree” and “I completely agree.” An increase in scores on the scale indicates higher levels of individuals’ life satisfaction. When the confirmatory factor analysis findings of the scale were analyzed with data collected from adults, it was found to have an acceptable level of fit (*χ*^2^/d*f* = 1.17, NFI = 0.99; GFI = 0.99; SRMR = 0.019; RMSEA = 0.030).

##### 3.1.2.3 UCLA loneliness scale-short form (ULS)

ULS was developed by Russell et al. (1978). This created scale has been revised and examined in cross cultural studies. Subsequently, an 8 item short form was created by Hays and DiMatteo (1987). Turkish adaptation of the scale was published by Doğan et al. (2011). The scale consists of eight items and has a one dimensional structure. The four point Likert type scale has options between “Never” and “Always.” The total score of all items means the overall loneliness score, and a high score indicates the severity of loneliness perceived by the individual. In the adaptation study, the scale’s Cronbach’s alpha internal consistency coefficient was calculated as 0.72.

#### 3.1.3 Data analysis

All data were analyzed with SPSS 26 and AMOS Graphics 24. Correlation tests were performed between variables. In the structural model created to test serial mediation, PRISM-TR was included in the model with its original four dimensional structure. Since the Bergen Social Media Addiction Scale (BSMAS), the Satisfaction with Life Scale (SLS), and the UCLA Loneliness Scale-Short Form (ULS) each had a unidimensional structure, two item parcels were created per scale. Mediation analyses were then conducted through structural equation modeling to explore the mediating effects of social media use and life satisfaction on the link between loneliness and parasocial relationships. Additionally, the significance of the indirect effects was tested using bootstrap procedures.

### 3.2 Results

First of all, the relationships between the variables in the study were examined. Descriptive statistics and correlation values of the variables are given in Table 6.

**Table 6:** Descriptive statistics and correlation.

Variables	1	2	3	4	Cronbach’s alpha	Mean	SD	Skewness	Kurtosis
1. BSMAS	—				0.82	18.71	5.29	−0.16	−0.42
2. ULS	0.41	—			0.88	14.24	5.37	0.95	0.21
3. SLS	−0.43	−0.54	—		0.81	14.67	3.65	0.09	−0.52
4. PRISM-TR	0.48	0.50	−0.46	—	0.97	46.78	19.11	0.64	−0.07

As seen in Table 6, all variables were found to be related to each other. Following the correlation analysis, the structural model was analyzed. Structural equation modeling findings showed that model fit was good (*χ*^2^*/*d*f* = 3.69; CFI = 0.982; GFI = 0.970; TLI = 0.972; RMSEA = 0.063). The results show that social media use and life satisfaction serially and partially mediate the relationship between loneliness and parasocial relationships. Table 7 presents the direct and indirect effects along with their 95% confidence intervals. The analysis revealed that the indirect effect was statistically significant (bootstrap estimate = 0.274, 95% CI = 0.211–0.346). These findings support the conclusion that social media use and life satisfaction significantly mediate the relationship between loneliness and parasocial relationships (see Table 7). The standardized path coefficients are illustrated in Figure 1.

**Table 7:** Standardized parameters and 95% CIs for direct and indirect effects.

Model pathways	Estimated	95% CI	
		Lower	Upper
Direct link			
Loneliness → social media use	0.490	0.407	0.564
Loneliness → life satisfaction	−0.500	−0.580	−0.416
Loneliness → parasocial relationships	0.284	0.171	0.390
Social media use → life satisfaction	−0.301	−0.393	−0.206
Social media use → parasocial relationships	0.354	0.254	0.448
Life satisfaction → parasocial relationships	−0.155	−0.259	−0.049
Indirect link			
Loneliness → social media use → life satisfaction → parasocial relationships	0.274	0.211	0.346

**Figure 1:**
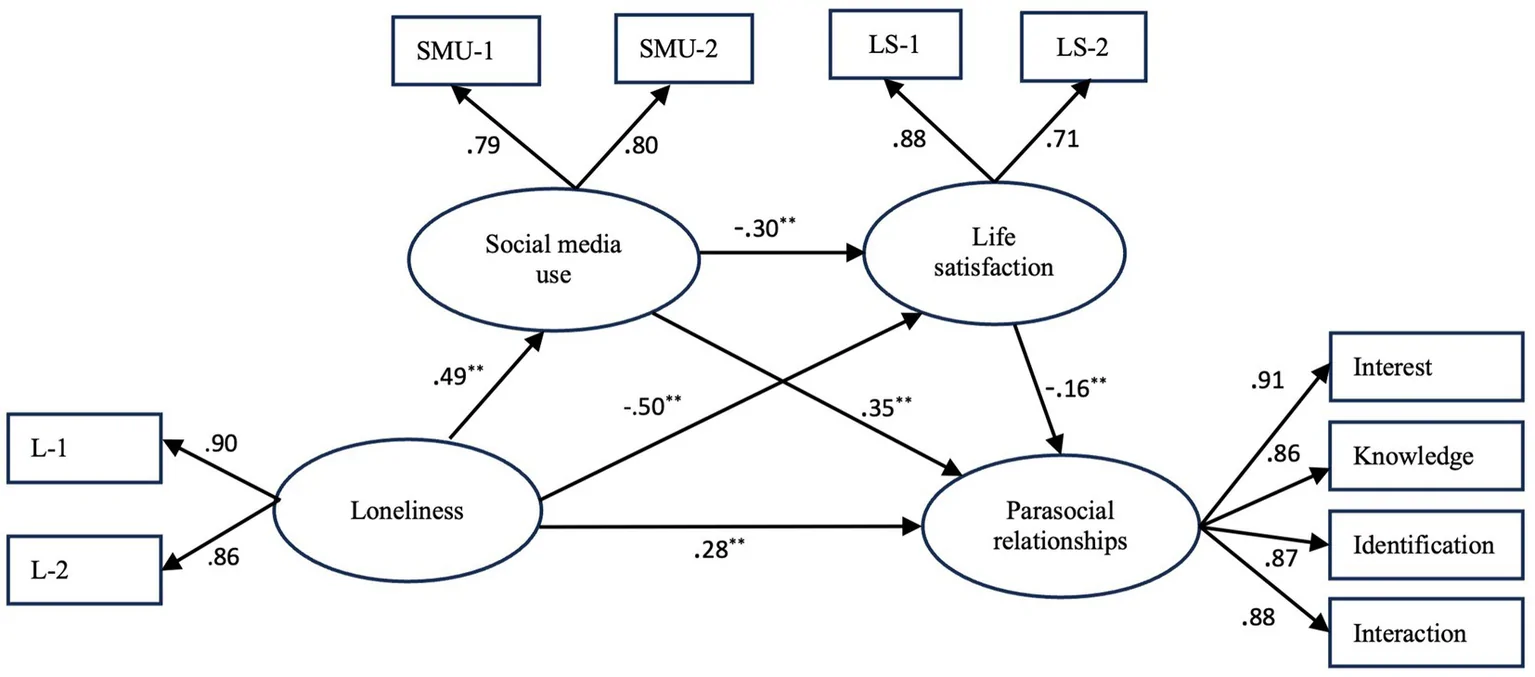
Structural equation model illustrating the serial mediating roles of social media use and life satisfaction in the relationship between loneliness and parasocial relationships. **p < 0.01.

## 4 Discussion

In this study, the PRISM scale, developed to measure parasocial relationships that emerge in the context of social media, was successfully adapted into Turkish. Accordingly, the first hypothesis of the study was confirmed (H1). The results of the confirmatory factor analysis indicated that the four factor structure of the original scale was retained in the Turkish sample. The obtained fit indices demonstrated a good fit of the model with the data, supporting the construct validity of the scale. Furthermore, the internal consistency coefficients of each sub-dimension were within acceptable levels, confirming the reliability of the scale. In addition, to examine whether the scale functioned equivalently for male and female participants, a measurement invariance analysis was conducted. The results showed that structural equivalence was valid between gender groups. Moreover, the scale showed clear unidimensionality, supported by favorable model fit indicators and robust item discrimination results from the IRT analysis. Discrimination coefficients (*α*) exceeding 1.0 highlighted the items’ strong ability to distinguish among individuals across the underlying construct (Baker and Kim, 2017). These findings indicate that the PRISM-TR scale can reliably and validly measure parasocial relationships between social media users and content creators in Turkish speaking samples.

Beyond confirming the factorial validity of the PRISM-TR scale, the present findings invite a substantive interpretation of how each dimension operates within the Turkish cultural context. Factor loadings exceeded those reported in the original validation study across all four dimensions, with this difference being most pronounced in the Identification with and Interaction with subscales. This pattern suggests that the dimensions capturing perceived similarity, value congruence, and a sense of companionship with media figures operate with greater psychometric precision in the Turkish sample. From a cultural standpoint, this finding may be interpreted in light of the collectivist orientation of Turkish society, which places strong emphasis on relational closeness, interpersonal harmony, and in-group belonging (Kağıtçıbaşı, 2007). In collectivist cultural contexts, individuals tend to define themselves in relation to significant others and derive a substantial part of their identity from perceived group memberships and shared values (Triandis, 1995). Within such a framework, the tendency to perceive media figures as psychologically proximate sharing one’s beliefs, attitudes, and ways of engaging with the world may be especially salient, rendering the Identification with and Interaction with dimensions particularly resonant for Turkish social media users. In Turkish culture, similarity and closeness are regarded as fundamental indicators of trustworthy relationships. This cultural inclination may facilitate social media users’ experiencing content creators as part of their own social circles particularly when shared values and attitudes are perceived. This cultural framework thus renders the elevated factor loadings observed in the Identification with and Interaction with dimensions more interpretable.

The Interest in and Knowledge Of dimensions also demonstrated strong factor loadings and high item discrimination in the Turkish sample, although the differences from the original study were relatively smaller for these dimensions. This pattern may be interpreted as indicating that these dimensions reflect more universal parasocial processes that operate relatively independently of cultural context. Indeed, cross cultural research on parasocial relationships has shown that the emotional and cognitive components of these bonds function largely consistently across different cultural settings, whereas relational and behavioral dimensions tend to be more sensitive to cultural context (da Costa Daniele et al., 2025; Schmid and Klimmt, 2011). The desire to follow a content creator’s posts and to actively seek information about them constitutes fundamental cognitive mechanisms of social media engagement, directly shaped by platform level features such as accessibility, visibility, and continuous content flow (Hoffner and Bond, 2022). In contrast, the more pronounced differences observed in the Identification with and Interaction with dimensions suggest that these subscales tap into deeper relational and identity based processes, rendering them more sensitive to cultural values. Although cross cultural comparisons in parasocial research remain limited in number (Schramm et al., 2024), existing evidence indicates that collectivist orientations strengthen parasocial bonding and that individuals in such cultures tend to exhibit more intense attachment tendencies (Du et al., 2025). This pattern of findings suggests that parasocial relationships constitute a multilayered structure, simultaneously drawing on universal media consumption dynamics and culturally specific relational processes.

The finding of measurement invariance across gender further reinforces the structural robustness of PRISM-TR, indicating that the four factor model functions equivalently for both male and female users in the Turkish context. This is particularly noteworthy given the distinct socialization patterns associated with gender in Turkish society (Sunar and Fişek, 2005), suggesting that the psychological mechanisms underlying parasocial relationship formation on social media transcend gender specific relational norms. Taken together, the Turkish adaptation may be said to not merely replicate the original factor structure, but to reflect culturally meaningful variation in how parasocial bonds are experienced, expressed, and sustained in a collectivist digital landscape.

Given the limited availability of Turkish measurement tools that address parasocial relationships within the conceptual framework of social media, the adaptation of the PRISM-TR scale fills an important gap in the literature. Existing Turkish scales tend to focus more on the influence of social media figures within the context of popular culture or adolescence. However, PRISM-TR provides a framework for examining parasocial relationships during emerging adulthood. Since emerging adulthood is a developmental period in which identity formation, the need for belonging, and social orientation become more salient, the availability of a valid and reliable tool for evaluating parasocial bonds in this phase is of great importance for both theoretical and applied research. Additionally, PRISM-TR conceptualizes parasocial relationships within the interactive nature of social media environments and allows for the measurement of this construct. In this respect, the scale enables a more holistic investigation of the psychological processes underlying users’ relationships with content creators, making it a valuable tool for applied research in social media studies, psychology, communication, and media disciplines.

Following the findings on construct validity and measurement invariance, the predictive validity of the PRISM-TR scale was examined by testing the extent to which the theoretically relevant variables predicted such connections. The link between loneliness and parasocial relationships plays a significant role in shaping individuals’ patterns of social interaction in the digital age. Exploring the mediating role of social media use and life satisfaction in this relationship is crucial for uncovering the dynamics that influence individuals’ psychological wellbeing. In line with the study’s hypotheses, the findings are discussed in detail in the following sections.

According to the second finding of the study, loneliness positively predicts parasocial relationships (H2). This result is consistent with previous research in the literature (Baek et al., 2013; Stein et al., 2024; Liu and Lee, 2024; Lotun et al., 2024). Existing studies suggest that parasocial relationships bear significant similarities to real life social relationships; however, there is still no clear consensus on the psychological mechanisms that govern these bonds. Madison et al. (2016) argued that under certain conditions, individuals may prefer parasocial connections over real life relationships, partially fulfilling their need to belong. This suggests that one-sided relationships may serve as a functional coping strategy associated with loneliness. When individuals do not derive sufficient satisfaction from real social connections, perceived social disconnection tends to align with their tendency to seek alternative forms of attachment. In this context, parasocial bonds can become a means of compensating for emotional needs such as support, a sense of belonging, and social approval among individuals experiencing social isolation (Giles and Naylor, 2000; Rubin and Perse, 1987; Horton and Wohl, 1956).

The propensity of lonely individuals to form parasocial relationships may not only be associated with an attempt to escape the feeling of loneliness but also reflect a desire to develop a sense of belonging and form meaningful connections (Ravi and Patki, 2025). Moreover, the inclination may also be fuelled by the need to compensate for negative emotions often associated with loneliness, such as sadness, emptiness, and feelings of worthlessness (Stein et al., 2024). Parasocial relationships established through social media, in particular, can serve a loneliness reducing function, as media figures often share aspects of their daily lives and create an impression of emotional intimacy with their followers (Stever, 2024; Giles and Stever, 2024).

Findings indicating that parasocial relationships tend to increase in contexts characterized by low social satisfaction and limited face to face interaction (e.g., Bond, 2021b; Hoffner and Bond, 2022; Lotun et al., 2024) support this result. Furthermore, in periods of heightened loneliness such as the widespread physical isolation of the pandemic non-reciprocal connections tend to become more prominent, functioning as a coping mechanism (Hoffner and Bond, 2022). Accordingly, the current finding suggests that loneliness may relate to a greater tendency toward parasocial bonds, which are perceived as safer, lower risk, and emotionally fulfilling alternatives, and that this inclination becomes even more visible in digital media environments.

The third result suggests that social media use serves as a mediator between loneliness and parasocial relationships (H3). The initial stage of this mediated relationship aligns with the Compensatory Internet Use Theory, which posits that individuals initially turn to digital platforms as a coping mechanism for feelings of loneliness, exclusion, or emotional distress (Kardefelt-Winther, 2014; Fioravanti et al., 2019). Empirical studies have shown that individuals who struggle to meet their social needs tend to use social media platforms more frequently, seek social stimulation through them, and report higher usage rates (Chura-Quispe et al., 2025; Ellison et al., 2007; Primack et al., 2017; Satici, 2019; Song et al., 2014; Wang et al., 2012; Wright and Riviera, 2025). This increased engagement may facilitate the development of parasocial relationships. Since social media offers instant interaction, visibility, and accessibility, it becomes an appealing tool for coping with feelings of isolation. In such digital environments, parasocial relationships formed with media figures may serve as alternative attachments that help compensate for low social satisfaction and foster a sense of belonging.

Liu and Lee (2024) showed that engaging with social media influencers is associated with parasocial connections, which relate to individuals’ feelings of belonging and correlate with their perception of social support, potentially relating to better coping with loneliness. Similarly, Hoffner and Bond (2022) found that during periods of increased physical isolation such as the COVID-19 pandemic social media use served as a tool for compensating for the lack of interpersonal interaction, which in turn was associated with more prominent parasocial relationships. These findings suggest that social media use mediates the link between loneliness and parasocial relationships by encouraging individuals to seek one-sided connections that address emotional needs caused by social disconnection.

The fourth finding of the study indicates that life satisfaction mediates the relationship between loneliness and parasocial relationships (H4). This result can be interpreted through the parasocial compensation hypothesis (Madison et al., 2016), which proposes that individuals who do not derive sufficient satisfaction from their real-life interactions may form one-sided bonds with media figures to compensate for unmet social and emotional needs. Rather than representing a mere by-product of digital interaction, lower life satisfaction in this model reflects a broader evaluation of one’s offline circumstances that may orient individuals toward such compensatory bonds. As noted in the introduction, life satisfaction is treated here as a global and comparatively stable cognitive evaluation (Diener et al., 1985), which supports positioning it as an antecedent of parasocial engagement rather than as its outcome. When individuals appraise their offline reality as unsatisfying in the context of persistent loneliness, parasocial bonds may offer accessible, low-risk, and emotionally safe connections through which they attempt to address these unmet needs, without the vulnerability associated with reciprocal face-to-face relationships.

As expected, social media use and life satisfaction were found to have a serial mediating effect on the relationship between loneliness and parasocial relationships (H5). In this regard, loneliness emerges as a key determinant of individuals’ orientation toward digital environments. Those who struggle to meet their social needs may increasingly turn to social media platforms to fill this void (Nowland et al., 2018). However, social media use is associated with lower life satisfaction alongside excessive time consumption, social comparison, fear of missing out (FOMO), and fewer of face-to-face interactions (Kross et al., 2013; Twenge et al., 2018; Verduyn et al., 2017; Vogel et al., 2014). When individuals experience decreased life satisfaction and are not fully satisfied with real life social bonds, they may seek alternative ways to fulfill their emotional needs such as support, belonging, and social approval. At this point, parasocial relationships offer a safe, accessible, and low risk form of social interaction. The accessibility of media figures, their intimate content sharing, and the absence of reciprocity expectations make these relationships particularly appealing (Stever, 2024; Labrecque, 2014). Ultimately, our findings provide evidence for a potential serial pathway where perceived social disconnection is associated with increased social media use and decreased life satisfaction, which in turn relate to engagement in parasocial relationships. Thus, social media use and life satisfaction may play significant roles as serial mediators in the link between loneliness and parasocial relationships.

## 5 Conclusion

In this study, the PRISM-TR scale developed to measure parasocial relationships within the context of social media was successfully adapted into Turkish, demonstrating structural validity, reliability, and measurement invariance across genders. In addition, the study proposed a comprehensive model to understand how individuals in the digital age fulfill their social and emotional needs by examining the relationships among loneliness, social media use, life satisfaction, and parasocial relationships. The results indicated that elevated feelings of loneliness can drive greater engagement with social media, which may subsequently diminish individuals’ life satisfaction. Decreased life satisfaction may then drive individuals to form more intense parasocial relationships with media figures. These results offer an integrative framework for understanding how the interplay of loneliness, social media use, and life satisfaction shapes the development of parasocial connections in digital environments. In the age of social media, digital interactions used to cope with negative emotions such as loneliness may transform parasocial relationships from passive viewing experiences into active and meaningful social bonds.

In conclusion, this study contributes to the literature both at the level of measurement and theoretical modeling. The Turkish adaptation of the PRISM-TR scale offers a psychometrically sound instrument for measuring parasocial relationships within social media environments, and the tested structural model contributes to a deeper understanding of how individuals meet their social needs online. Therefore, the findings of this study lay a foundation for developing new research areas and intervention strategies in the fields of psychological counseling, clinical practice, and social media studies.

### 5.1 Limitations and suggestions for future research

Although the present study provides valuable contributions to the literature by adapting the PRISM scale into Turkish and testing a comprehensive structural model, it is not without limitations. Despite the rigorous psychometric testing, the exclusive use of university student samples remains a significant limitation that restricts the generalizability of the findings to the broader Turkish population. While measurement invariance was established across gender, this study did not explore other potential sources of heterogeneity, such as socioeconomic status, education level, or relationship status, which might influence the formation of parasocial bonds. Future research should incorporate more diverse demographic groups and test for invariance across these variables to ensure the scale’s universal applicability.

Besides, the study’s cross-sectional nature restricts the capacity to establish causal links among loneliness, social media use, life satisfaction, and parasocial relationships. It is important to acknowledge that the directional pathways proposed from loneliness to parasocial engagement via social media use represent only one plausible theoretical sequence. Alternative explanations, such as a bidirectional relationship where intense parasocial engagement might lead to increased social withdrawal and subsequent loneliness, cannot be ruled out. Consequently, the reported associations should be interpreted as a snapshot of complex psychological interrelations rather than a confirmed causal chain. Longitudinal or experimental research designs are recommended for future studies to capture the temporal dynamics and causal pathways of these relationships more effectively.

Second, while the adaptation of the PRISM-TR scale followed rigorous psychometric procedures including CFA and reliability indices, the scale’s ecological validity in different digital platforms (e.g., TikTok vs. YouTube vs. Instagram) was not assessed. Given the diversity of social media platforms and user interaction styles, future research should explore whether parasocial relationships differ meaningfully across platforms and adapt or validate the instrument accordingly. Additionally, because the survey instruments were administered in a fixed, non-counterbalanced order, the potential for order or fatigue effects cannot be ruled out. Future studies should employ block randomization or counterbalancing procedures during data collection to mitigate these methodological biases.

Another important limitation of the present study concerns the absence of qualitative data regarding the nature, targets, and motivations of participants’ parasocial relationships. While the PRISM-TR scale effectively captures the intensity and multidimensional structure of parasocial bonds, it does not assess who the parasocial relationships were formed with or why specific content creators were chosen. This represents a limitation not only of the present study but also of the broader parasocial relationship literature, in which the vast majority of research has relied on surveys and existing measurement instruments (Schramm et al., 2024). Although a limited number of qualitative and mixed methods studies have begun to explore participants’ own accounts of their parasocial motivations revealing that such relationships may be driven by admiration of the media figure’s characteristics and content quality rather than solely by compensation for social deficits (Ravi and Patki, 2025) these findings have yet to be integrated into scale based research. Future research would benefit from adopting mixed methods designs that complement PRISM-TR scores with semi-structured interviews or open ended questions, thereby elucidating the subjective meanings, motivations, and experiential dimensions of parasocial relationships among Turkish young adults. Such an approach would enable an understanding of why specific media figures are chosen as parasocial targets and how these relationships function within the daily lives of emerging adults in their cultural context.

### 5.2 Theoretical and practical implications

The present study contributes to the literature on two distinct levels: measurement and theoretical modeling. At the measurement level, the successful Turkish adaptation of the PRISM-TR scale provides researchers with a psychometrically sound instrument for assessing parasocial relationships within social media contexts. The retention of the original four factor structure in a collectivist cultural context is itself theoretically meaningful: this finding suggests that the fundamental architecture of parasocial relationships, as captured by the PRISM-TR, may be sufficiently robust to transcend cultural boundaries, while the elevated factor loadings observed in the Identification With and Interaction With dimensions indicate that the relational and identity based aspects of these bonds may be experienced with particular salience in collectivist settings. This pattern of findings may encourage scholars working in similarly collectivist contexts to treat cultural orientation as a potential moderating variable in parasocial relationship research, rather than approaching such relationships as culturally invariant phenomena.

The study’s second level of contribution pertains to theoretical modeling. The serial mediation findings demonstrate how the foundational premise of the Compensatory Internet Use Theory can be integrated into a broader psychological framework, moving beyond the theory’s conventional focus on problematic internet use. Rather than altering the theory itself, the present findings suggest that initial compensatory digital engagement can serve as a catalyst within a wider sequential process that ultimately encompasses parasocial relationship formation. The sequential pattern of associations observed among loneliness, social media use, life satisfaction, and parasocial involvement suggests that parasocial relationship formation may be less a direct response to loneliness than the outcome of a more complex process involving digital engagement and subjective wellbeing. The emergence of social media use and life satisfaction as serially operating intermediate mechanisms in this chain contributes to addressing the gap noted in the introduction regarding the limited development of integrative models that simultaneously encompass all four variables.

From an applied standpoint, the findings carry tentative implications for practitioners working with university students, though the cross-sectional design of the study warrants caution in translating these results into direct practice recommendations. Practitioners in university counseling settings may therefore find it meaningful to consider loneliness, social media use, and life satisfaction in conjunction for instance, by exploring students’ experiences of loneliness and subjective wellbeing alongside their patterns of social media engagement rather than addressing each variable in isolation (Nowland et al., 2018; Hoffner and Bond, 2022). In this context, Diaz et al. (2026) suggest that incorporating parasocial relationship awareness into broader social media screenings may represent a clinically relevant step.

## Data Availability

The original contributions presented in the study are included in the article/Supplementary material, further inquiries can be directed to the corresponding author.

## Author’s note

This study was presented as an oral presentation at the 27th International Congress on Psychological Counseling and Guidance, held on June 11, 2026, in Konya, Türkiye.
