# Supplementary material for: Adaptation of Parasocial Relationships in Social Media Scale: its association with loneliness, social media use and life satisfaction
Source: Front Psychol. 2026 Sep 17;17:1814389. doi: 10.3389/fpsyg.2026.1814389 (PMC13627288; doi:10.3389/fpsyg.2026.1814389)
Supplement: Supplementary file 1 [file Data_Sheet_1.pdf]

Appendix A: Online Supplement - Sample Item Translation Process

To ensure semantic equivalence between the original English Parasocial Relationships in Social Media (PRISM) scale and its Turkish adaptation, a rigorous back-translation procedure was followed (Brislin, 1970). Table S1 illustrates this process using the 4th item of the scale.

Table S1.

An illustration of the translation and back-translation process.

Step	Language	Sample Item (Interest Dimension - Item 4)
**Original Item**	English	I care about what happens to [NAME].
**Initial Translation**	Turkish	Ona ne olduğunu önemsiyorum.
**Back-Translation**	English	I care about what happens to him/her.
**Final Decision**	-	The back-translation matched the original item perfectly, confirming semantic and contextual equivalence. The Turkish translation was retained without modification.
