# Supplementary material for: Adaptation of Parasocial Relationships in Social Media Scale: its association with loneliness, social media use and life satisfaction
Source: Front Psychol. 2026 Sep 17;17:1814389. doi: 10.3389/fpsyg.2026.1814389 (PMC13627288; doi:10.3389/fpsyg.2026.1814389)
Supplement: Supplementary file 2 [file Data_Sheet_2.pdf]

SOSYAL MEDYADA PARASOSYAL İLİŞKİLER ÖLÇEĞİ (PRISM-TR)

Lütfen aşağıdaki ifadeleri dikkatlice okuyunuz ve takip ettiğiniz/ilgi duyduğunuz sosyal medya figürünü düşünerek size en uygun olan seçeneği işaretleyiniz. Doğru veya yanlış cevap yoktur.

1: Kesinlikle katılmıyorum, **2**: Katılmıyorum, **3**: Ne katılıyorum ne katılmıyorum, **4**: Katılıyorum
5: Kesinlikle katılıyorum

		1	2	3	4	5
1	Onun içeriklerini görmeyi dört gözle beklerim.					
2	Onun yaptığı içerikleri görmek hoşuma gider.					
3	Ona ilgi duyuyorum.					
4	Ona ne olduğunu önemsiyorum.					
5	Umarım o hedeflerine ulaşır.					
6	Eğer onu takip ettiğim sosyal medya platformunda başka bir hesap açarsa, o hesabını da takip ederim.					
7	Onun içinde yer aldığı ama kendi oluşturmadığı içerikleri görmek hoşuma gidiyor.					
8	Onun hakkında bilgiye rastladığımda, daha fazlasını öğrenmek için araştırırım.					
9	Onun hakkında daha fazlasını öğrenmek için araştırırım.					
10	Onun hakkında bilgi sahibi olmak benim için önemli.					
11	Onun hayatıyla ilgili kişisel ayrıntıların farkındayım.					
12	Eğer onunla ilgili bir hikâye (story) görürsem, izlerim.					
13	Onunla benzer inançlara sahibim.					
14	Onunla benzer düşüncelere sahibim.					
15	Genelde onunla aynı fikirde olurum.					
16	Genellikle onunla aynı seçimleri yaparım.					
17	Onun tutumlarını kendime yakın buluyorum.					
18	Onun sorunları ele alma biçimini severim.					
19	O bana bir arkadaşımla birlikteymişim gibi hissettiriyor.					
20	İçeriklerine bakarken o bana arkadaşlık ediyor.					
21	Kendimi onun arkadaşı olarak hayal edebiliyorum.					
22	O, benim görmek istediğim içerik çeşitlerini anlayabiliyor.					

Puanlama ve Alt Boyutlar (Scoring and Subscales):

Ölçekte ters kodlanan (reverse-scored) madde bulunmamaktadır.

- İlgi (Interest): 1, 2, 3, 4, 5, 6, 7
- Bilgi (Knowledge): 8, 9, 10, 11, 12
- Özdeşim Kurma (Identification): 13, 14, 15, 16, 17, 18

- Etkileşim (Interaction): 19, 20, 21, 22

Orijinal Ölçek (Original Scale): Boyd, A. T., Morrow, J. A., & Rocconi, L. M. (2022). Development and validation of the Parasocial Relationship in Social Media survey. *The Journal of Social Media in Society*, *11*(2), 192-208.

Türkçe Uyarlama (Turkish Adaptation): Bu Türkçe form kullanıldığında lütfen aşağıdaki çalışmaya atıf yapınız. / Please cite the following study when using this Turkish adaptation.

Kurt, I. E., Bulut-Ateş, F., & Doğanülkü, H. A (2026). Adaptation of Parasocial Relationships in Social Media Scale: Its Association with Loneliness, Social Media Use and Life Satisfaction. *Frontiers in Psychology*, *17*, 1814389.
